# Nitrogen-Doped Carbon-Encased Bimetallic Selenide for High-Performance Water Electrolysis

**DOI:** 10.1007/s40820-019-0299-4

**Published:** 2019-08-08

**Authors:** Junhui Cao, Kexin Wang, Jiayi Chen, Chaojun Lei, Bin Yang, Zhongjian Li, Lecheng Lei, Yang Hou, Kostya Ostrikov

**Affiliations:** 1 Key Laboratory of Biomass Chemical Engineering of Ministry of Education, College of Chemical and Biological Engineering, Zhejiang University, Hangzhou, 310058 People’s Republic of China; 2 Ningbo Research Institute, Zhejiang University, Ningbo, 315100 People’s Republic of China; 3 Institute of Zhejiang University - Quzhou, 78 Jiuhua Boulevard North, Quzhou, 324000 People’s Republic of China; 4 School of Chemistry, Physics, and Mechanical Engineering, Queensland University of Technology, Brisbane, QLD 4000 Australia

**Keywords:** Core–shell structure, Bimetallic selenide, N-doped carbon, Synergistic effect, Oxygen evolution reaction

## Abstract

Demand of highly efficient earth-abundant transition metal-based electrocatalysts to replace noble metal materials for boosting oxygen evolution reaction (OER) is rapidly growing. Herein, an electrochemically exfoliated graphite (EG) foil supported bimetallic selenide encased in N-doped carbon (EG/(Co, Ni)Se_2_–NC) hybrid is developed and synthesized by a vapor-phase hydrothermal strategy and subsequent selenization process. The as-prepared EG/(Co, Ni)Se_2_–NC hybrid exhibits a core–shell structure where the particle diameter of (Co, Ni)Se_2_ core is about 70 nm and the thickness of N-doped carbon shell is approximately 5 nm. Benefitting from the synergistic effects between the combination of highly active Co species and improved electron transfer from Ni species, and N-doped carbon, the EG/(Co, Ni)Se_2_–NC hybrid shows remarkable electrocatalytic activity toward OER with a comparatively low overpotential of 258 mV at an current density of 10 mA cm^−2^ and a small Tafel slope of 73.3 mV dec^−1^. The excellent OER catalysis performance of EG/(Co, Ni)Se_2_–NC hybrid is much better than that of commercial Ir/C (343 mV at 10 mA cm^−2^ and 98.1 mV dec^−1^), and even almost the best among all previously reported binary CoNi selenide-based OER electrocatalysts. Furthermore, in situ electrochemical Raman spectroscopy combined with ex situ X-ray photoelectron spectroscopy analysis indicates that the superb OER catalysis activity can be attributed to the highly active Co–OOH species and modified electron transfer process from Ni element.

**Electronic supplementary material:**

The online version of this article (10.1007/s40820-019-0299-4) contains supplementary material, which is available to authorized users.

## Highlights

- N-Doped carbon-encased bimetallic selenide hybrid with strong synergistic effect is constructed.
- N-Doped carbon-encased bimetallic selenide hybrid exhibits an excellent catalytic activity and stability for oxygen evolution reaction (OER) in alkaline media.
- The superior OER activity is attributed to the highly active Co–OOH species and modified electron transfer process from Ni atoms.

**Electronic supplementary material:**

The online version of this article (10.1007/s40820-019-0299-4) contains supplementary material, which is available to authorized users.

## Graphical abstract

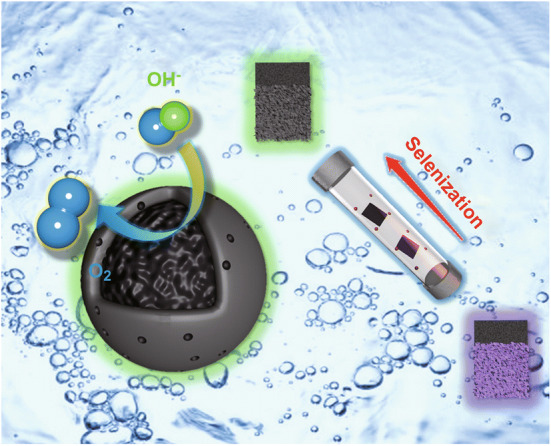

## Introduction

Reliable and sustainable supply of clean energy has recently emerged as a critical socioeconomic megatrend [1–4]. Hydrogen, an ideal carbon-free energy source, with high heat value and environment-friendly characteristics, can be produced by electrochemical water splitting. In whole electrochemical reaction, hydrogen evolves from the proton reduction at the cathode by a facile two-electron transfer procedure. However, a much more sluggish oxygen evolution reaction (OER) with a four-electron transfer process from water oxidation dominates the reaction kinetics of water splitting and impedes the desired fast hydrogen evolution [5, 6]. Therefore, it is pivotal to design highly efficient electrocatalysts suitable to boost the water oxidation process. Noble metal (e.g., Ir and Ru)-based anode electrocatalysts have proved their high activity in stimulating the OER performance [5, 7]. However, the noble metals are scarce while their stability under harsh electrolysis conditions needs substantial improvement, which limits their widespread utilization. This is why major efforts have been devoted worldwide to developing earth-abundant transition metal-based electrocatalysts, such as transition metal hydroxides (Co hydroxide, Ni–Fe hydroxide) [8–11], oxides (Co_3_O_4_, MoO_2_) [12, 13], sulfides (CoS_2_, Ni_3_S_2_) [5, 14], selenides (CoSe_2_, NiSe_2_) [15, 16], carbides (Mo_2_C, MoC_2_) [17, 18], and several others. However, single transition metal-based electrocatalysts suffer from several issues such as moderate activity and poor stability, which prevent these materials from commercial electrolysis applications [4, 19].

In recent years, binary metal or multi-metal selenides have emerged as promising water splitting electrocatalysts [20, 21]. Different from single transition metal catalysts, the electronic structure of binary transition metal electrocatalysts could be effectively improved via the introduction of secondary metal element with well-matched electronic configuration [22]. As a result, more exposed active sites and more facile electron transfer process can be achieved to enhance the electrocatalytic performance [23]. However, low electronic conductivity of current binary transition metals selenides still impedes the practical developments of bimetallic selenide-based energy materials [24, 25]. Previous research has indicated nitrogen doping into nanocarbon that can effectively modulate the electronic properties of hybrid catalysts involving N-doped carbon, eventually improving the overall water electrolysis performance [26].

Herein, we report a novel core–shell-structured binary metal selenide hybrid electrocatalyst consisting of penroseit (cobalt, nickel) selenide core and N-doped carbon shell supported on the electrochemically exfoliated graphite (EG) foil (EG/(Co, Ni)Se_2_–NC). The hybrid catalyst was fabricated by using a selenization treatment of CoNi-layered double hydroxide (CoNi–LDH)/zeolite imidazolate framework-67 (ZIF-67) nanosheet array. The nanosheet array was prepared by vapor-phase hydrothermal growth of ZIF-67 on the surface of CoNi–LDH nanosheets array. In this (Co, Ni)Se_2_–NC hybrid, the shell was composed by N-doped carbon with thickness of ~ 5 nm from the pyrolysis of 2-methylimidazole (2-MIm) in ZIF-67, while the core was composed by penroseit (Co, Ni)Se_2_ with particle diameter of ~ 70 nm, formed through the CoNi–LDH’s selenization. Owing to the synergistic effects of binary metal Co and Ni, as well as N-doped carbon, the EG/(Co, Ni)Se_2_–NC hybrid displayed a remarkable performance toward the OER electrocatalysis with a low overpotential of 258 mV at the current density of 10 mA cm^−2^, and a small Tafel slope of 73.3 mV dec^−1^, which was superior to commercial Ir/C and most reported binary CoNi selenide-based materials. In situ electrochemical Raman spectroscopy and ex situ X-ray photoelectron spectroscopy (XPS) results revealed the conversion of active species from Co–Se to Co–OOH species and confirmed the role of introduced Ni species in tuning its electronic structure by accelerating the electron transfer process.

## Experimental Section

### Synthesis of EG

Graphite foil was firstly rinsed by ultrapure water for three times and further dried at 60 °C for 1 h. Then, EG substrate was obtained with the method of electrochemical exfoliation in 0.05 M (NH_4_)_2_SO_4_ solution at 10 V for 3 min [27].

### Synthesis of EG/CoNi–LDH

0.4366 g of Co(NO_3_)_2_·6H_2_O and 0.2908 g of Ni(NO_3_)_2_·6H_2_O were dissolved in 50 mL ultrapure water. EG was set as the working electrode, carbon rod as counter electrode, and Ag/AgCl electrode as reference electrode, respectively. A typical three-electrode system was conducted to electrodeposit the CoNi–LDH on EG substrate at − 1.2 V (vs. Ag/AgCl) for 150 s. The final product of EG/CoNi–LDH was dried at 60 °C for 1 h.

### Synthesis of EG/CoNi–LDH/ZIF-67

The EG/CoNi–LDH/ZIF-67 hybrid was synthesized by a convenient vapor-phase hydrothermal strategy following previous report [27]. In this process, the EG/CoNi–LDH was supported on a Teflon pillar in the Teflon-lined autoclave, while 2-methylimidazole ligand was placed in the bottom of autoclave surrounding the pillar. This system was maintained at 150 °C for 20 min to form the EG/CoNi–LDH/ZIF-67.

### Synthesis of EG/(Co, Ni)Se_2_–NC

The as-prepared EG/CoNi–LDH/ZIF-67 and Se powder were put into a quartz tube and sealed in vacuum. Then, the tube was heated to different reaction temperatures (400, 500, and 600 °C) for 2 h. The CoNi–LDH was reacted with Se powder and then converted into (Co, Ni)Se_2_, while the ligand of ZIF-67 was pyrolyzed to N-doped carbon simultaneously.

### Synthesis of EG/CoSe-NC

Co(OH)_2_ nanosheet array was electrochemically deposited on EG foil in 0.05 M Co(NO_3_)_2_ solution. EG was set as the working electrode, carbon rod as counter electrode, and Ag/AgCl electrode as reference electrode, respectively. A typical three-electrode system was conducted to electrodeposit the Co(OH)_2_ on EG substrate at − 1.2 V (vs. Ag/AgCl) for 150 s. The final product of EG/Co(OH)_2_ was dried at 60 °C for 1 h. After the selenization process, the EG/CoSe-NC was obtained.

### Synthesis of EG/Ni_3_Se_2_

Ni(OH)_2_ nanosheet array was electrochemically deposited on EG foil in 0.05 M Ni(NO_3_)_2_ solution. EG was set as the working electrode, carbon rod as counter electrode, and Ag/AgCl electrode as reference electrode, respectively. A typical three-electrode system was conducted to electrodeposit the Ni(OH)_2_ on prepared EG substrate at − 1.2 V (vs. Ag/AgCl) for 150 s. The final product of EG/Ni(OH)_2_ was dried at 60 °C for 1 h. After the selenization process, the EG/Ni_3_Se_2_ was obtained.

### Characterization

Field-emission scanning electron microscope (FESEM, Hitachi SU-8010) and transmission electron microscope (TEM, JEM-2100F) were applied to observe the microscopic morphology of catalysts. X-ray diffraction (XRD, PANalytical) was tested to characterize the crystal structure of catalysts. Energy-dispersive X-ray spectroscopy (EDX, Oxford X-max80) which is equipped on FESEM was employed to verify the actual composition of catalysts at different stage. X-ray photoelectron spectroscopy (XPS, Thermo Fisher Scientific, Escalab 250Xi) was utilized to analyze the valence of major elements with Al Kα radiation. Raman spectroscopy (LabRAM HR Evolution) was measured in situ for explaining the alteration of catalysts during the OER.

### Electrochemical Measurements

All electrochemical measurements were evaluated at room temperature using the electrochemical analyzer (CHI 760E) in a typical three-electrode configuration where the carbon rod worked as counter electrode and the Ag/AgCl electrode performed as reference electrode. Before testing, the catalysts were cycled in the potential range from 0 to 0.8 V versus Ag/AgCl until a steady CV curve was observed. Linear sweep voltammetry (LSV) was performed at the scan rate of 5 mV s^−1^ to estimate the electrocatalytic activities. Both chronoamperometric and chronopotentiometric treatments were performed to measure the durability of catalysts. The frequency of electrochemical impedance spectroscopy (EIS) was arranged from 100 K to 0.01 Hz. Electrochemically active surface area (ECSA) was measured from the electrochemical double-layer capacitance (*C*_d1_) by analyzing CV curves at various scan rates (10, 20, 30, 40, 60, 80, and 100 mV s^−1^). All measured potentials in 1.0 M KOH electrolyte were converted to the reversible hydrogen electrode (RHE) via the Nernst equation (*E*_RHE_ = *E*_Ag/Ag/Cl_ + 0.059 × pH + 0.197). All polarization curves were calibrated with iR-correction with the following equation: *E*_final_ = *E*_0_ − (*i*R) V, in which *E*_0_ is the potential referenced to the polarization curves, *i* is the current at *E*_0_ and *R* results from the EIS figures.

## Results and Discussion

**Fig. 1:**
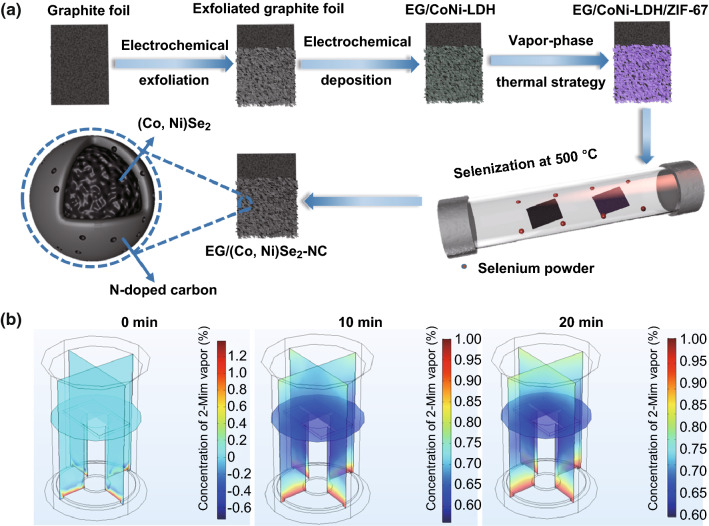
**a** Synthetic scheme of EG/(Co, Ni)Se_2_–NC hybrid and **b** simulation of 2-MIm evaporation and diffusion process

Figure 1a illustrates the synthesis procedure to obtain EG/(Co, Ni)Se_2_–NC hybrid. First, the CoNi–LDH nanosheet array was electrochemically deposited on the EG foil in Co(NO_3_)_2_ and Ni(NO_3_)_2_ mixed electrolyte. The obtained EG/CoNi–LDH was further converted into EG/CoNi–LDH/ZIF-67 nanosheet arrays by a vapor-phase hydrothermal treatment. In the hydrothermal vessel, solid 2-MIm powder was evaporated, leading to the formation of vapor-phase 2-MIm; then, the coordination reaction between the vaporized 2-MIm ligand and Co coordination center occurred, thus resulting in the formation of EG/CoNi–LDH/ZIF-67. Followed by further selenization treatment at 500 °C, penroseit (Co, Ni)Se_2_ core was obtained from the selenization of CoNi–LDH, and N-doped carbon shell was formed from the pyrolysis of 2-MIm ligand.

A simulation describing the concentration change of 2-MIm vapor was carried out to provide further understanding to the vapor-phase hydrothermal reaction process (Fig. 1b). Coupling the heat transfer model and diffusion model in COMSOL Multiphysics, we simulated the vapor-phase hydrothermal reaction process by analyzing the partial pressure (%) of 2-MIm vapor in this hydrothermal vessel. It was observed that at the beginning of heating (0 min), no 2-MIm vapor was found on the top of EG/CoNi–LDH. However, when the hydrothermal vessel was continuously heated, solid 2-MIm powder was evaporated and diffused to everywhere of this vessel (10 and 20 min). The coordination reaction between 2-MIm vapor and Co atoms in EG/CoNi–LDH contributed to the growth of ZIF-67, thus leading to the formation of EG/CoNi–LDH/ZIF-67 nanosheet arrays.

**Fig. 2:**
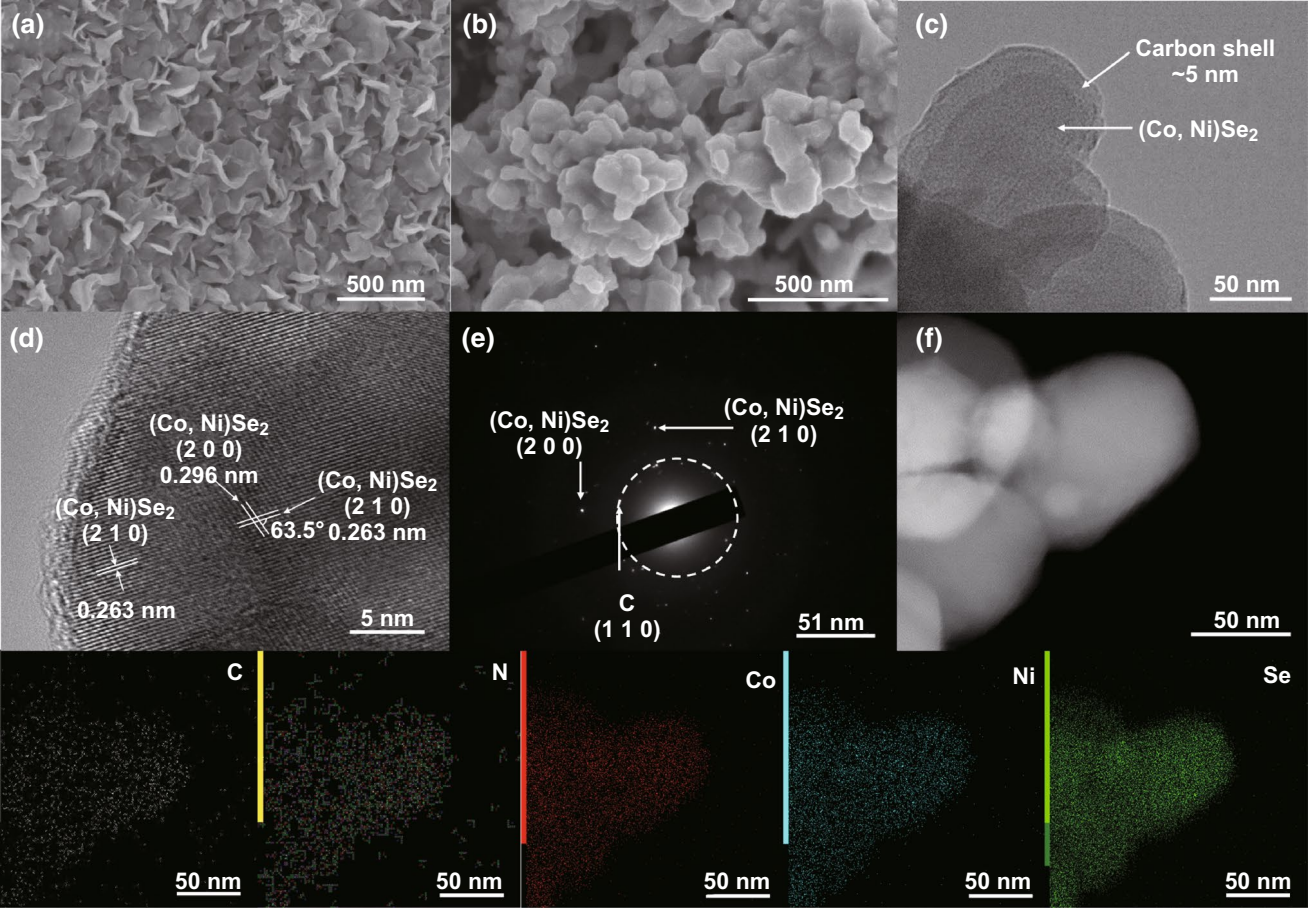
**a**, **b** FESEM images of EG/CoNi–LDH and EG/(Co, Ni)Se_2_–NC. **c, d** TEM and HRTEM images of EG/(Co, Ni)Se_2_–NC. **e** SAED pattern of EG/(Co, Ni)Se_2_–NC. **f** EDX and elemental mapping of analyzed elements: C, N, Co, Ni, and Se

The microstructure of as-prepared EG/(Co, Ni)Se_2_–NC hybrid was analyzed by a field-emission scanning electron microscopy (FESEM). Figure 2a, b shows the contrast of EG/CoNi–LDH/ZIF-67 and EG/(Co, Ni)Se_2_–NC’s morphology. The vertically flower-shaped layered structure of CoNi–LDH/ZIF-67 nanosheet arrays is clearly observed in Fig. 2a, which was similar to that of CoNi–LDH (Fig. S1, S2). After selenization treatment of EG/CoNi–LDH/ZIF-67, the EG/(Co, Ni)Se_2_–NC hybrid was obtained with particle size of about 80 nm piling on the surface of EG. Figure 2c showed the inner construction of EG/(Co, Ni)Se_2_–NC via transmission electron microscopy (TEM), where a core–shell structure composed of N-doped carbon shell with ~ 5 nm thickness and inner (Co, Ni)Se_2_ core with ~ 70 nm diameter was displayed. The N-doped carbon shell was derived from the pyrolysis of 2-MIm, while inside CoNi–LDH was reacted with Se source to form (Co, Ni)Se_2_ core. The high-resolution TEM (HRTEM) image of EG/(Co, Ni)Se_2_–NC hybrid revealed the lattice fringes of 0.263 and 0.296 nm (Fig. 2d), which could be well indexed to the (210) and (200) planes of (Co, Ni)Se_2_. It is notable that the angle measured between (210) and (200) planes was 63.5°, which was exactly the geometric angle between the two planes, indicating the existence of penroseit (Co, Ni)Se_2_ in EG/(Co, Ni)Se_2_–NC [2, 20, 28]. The selected area electron diffraction (SAED) pattern revealed the (210) and (200) planes of (Co, Ni)Se_2_ and the (110) plane of carbon (Fig. 2e), further confirming the co-existence of N-doped carbon shell and (Co, Ni)Se_2_ core in EG/(Co, Ni)Se_2_–NC hybrid. Energy-dispersive X-ray spectrum (EDX) and elemental mapping images showed that all the C, N, Co, Ni, and Se elements were well distributed in the EG/(Co, Ni)Se_2_–NC (Fig. 2f), revealing the existence of detected elements in this core–shell-structured (Co, Ni)Se_2_–NC.

**Fig. 3:**
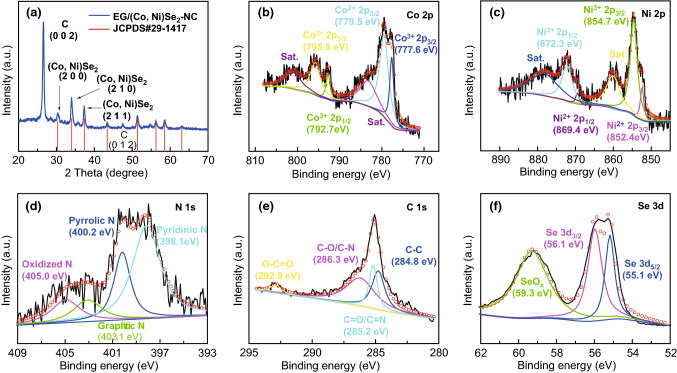
**a** XRD pattern of EG/(Co, Ni)Se_2_–NC. **b**–**f** high-resolution XPS spectra of Co 2*p*, Ni 2*p*, N 1*s*, C 1*s*, and Se 3*d* in EG/(Co, Ni)Se_2_–NC

X-ray diffraction (XRD) pattern of EG/(Co,Ni)Se_2_–NC is displayed in Fig. 3a. At 26.6°, a strong peak was indexed to the (002) plane of carbon. The other peaks located at 30.3°, 34.0°, 37.4°, 42.7°, 50.6°, 55.3°, 57.6°, and 62.0° represented the (200), (210), (211), (220), (311), (230), (321), and (400) planes of penroseit (Co, Ni)Se_2_, respectively (JCPDS No. 29-1417) [21], confirming that the cobalt precursor was thermally selenized into penroseit (Co, Ni)Se_2_. The full-range XPS spectrum evidenced the presence of C, N, O, Co, Ni, and Se elements in EG/(Co, Ni)Se_2_–NC hybrid (Fig. S3). In high-solution Co 2*p* XPS spectra (Fig. 3b), the Co with different chemical valances could be found, where the Co^3+^ 2*p*_3/2_ and Co^3+^ 2*p*_1/2_ were located at 777.6 and 792.7 eV, while the location of Co^2+^ 2*p*_3/2_ and Co^3+^ 2*p*_1/2_ were at 779.5 and 795.8 eV, respectively, in accordance with previously reported penroseit (Co, Ni)Se_2_ [2]. In high-solution Ni 2*p* XPS spectra (Fig. 3c), the situation of Ni was similar to that of Co, in addition to the different locations of each peak being at 854.7 eV for Ni^3+^ 2*p*_3/2_, 852.4 eV for Ni^2+^ 2*p*_3/2_, 872.3 eV for Ni^3+^ 2*p*_1/2_, and 869.4 eV for Ni^2+^ 2*p*_1/2_ [24, 29]. Deconvolution into high-resolution N 1*s* XPS spectra (Fig. 3d) gave the information of N species in N-doped carbon of EG/(Co, Ni)Se_2_–NC. Four different kinds of N species were analyzed as pyridinic N at 398.1 eV, pyrrolic N at 400.2 eV, graphitic N at 403.1 eV, and oxidized N at 405.0 eV, respectively. The existence of C–N bonds could be further evidenced in high-resolution C 1*s* XPS spectra (Fig. 3e). Except for C–C peak at 284.8 eV and O–C=O peak at 292.9 eV, the carbon atoms in the form of C–N and C=N bonds could be found at 286.3 and 285.2 eV, respectively. These results indicated the formation of N-doped carbon in the EG/(Co,Ni)Se_2_–NC. One doublet located at 55.1 and 56.1 eV could be found in high-resolution Se 3*d* XPS spectra and well indexed to correspondent characteristic peaks of Se 3*d*_5/2_ and Se 3*d*_3/2_ in the peronseit (Co, Ni)Se_2_. Besides, the SeO_*x*_ that resulted from the account of oxygen absorption is observed in Fig. 3f [20, 30].

**Fig. 4:**
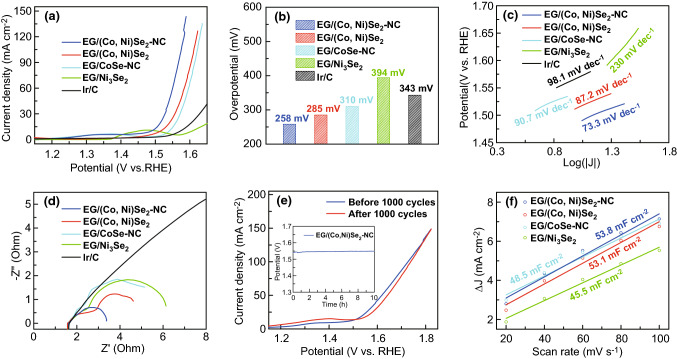
**a** Polarization curves, **b** overpotential plots at 10 mA cm^−2^, **c** Tafel slopes, **d** EIS spectra of EG/(Co, Ni)Se_2_–NC, EG/(Co, Ni)Se_2_, EG/CoSe-NC, EG/Ni_3_Se_2_, and Ir/C. **e** Polarization curves of EG/(Co, Ni)Se_2_–NC before and after CV cycles scanning. inset: chronoamperometric *E*-*t* plot at 20 mA cm^−2^. **f** *C*_dl_ plots of EG/(Co, Ni)Se_2_–NC, EG/(Co, Ni)Se_2_, EG/CoSe-NC, and EG/Ni_3_Se_2_

The electrocatalytic activity of EG/(Co,Ni)Se_2_–NC for OER was performed in 1.0 M KOH solution within a classic three-electrode system. The polarization curve (Fig. 4a) showed that the EG/(Co, Ni)Se_2_–NC hybrid owned the most powerful effect to catalyze OER with the lowest overpotential of 258 mV at the current density of 10 mA cm^−2^, while the overpotentials at the same current density were 285 mV for EG/(Co, Ni)Se_2_, 310 mV for EG/CoSe-NC, 394 mV for EG/Ni_3_Se_2_, and 343 mV for commercial Ir/C, respectively (Fig. 4b). Contrasting EG/(Co, Ni)Se_2_–NC hybrid and EG/(Co, Ni)Se_2_, the lower overpotential in EG/(Co, Ni)Se_2_–NC hybrid implied that the N-doped carbon could efficiently improve the OER electrocatalysis by the synergistic effect. Moreover, the benefit from binary metals could be obviously evidenced by comparing bimetal selenide of EG/(Co, Ni)Se_2_–NC hybrid to single metal selenides of EG/Ni_3_Se_2_ and EG/CoSe-NC. Due to the synergy between binary metals Co and Ni, the OER performance of EG/(Co, Ni)Se_2_–NC hybrid was superior to that of single metal EG/CoSe-NC and EG/Ni_3_Se_2_, respectively, indicating that the EG/(Co, Ni)Se_2_–NC hybrid performed excellently for boosting OER. Moreover, the current density of EG/(Co, Ni)Se_2_–NC hybrid was 62.1 mA cm^−2^ at 1.55 V, which was 35.5 and 49.1 mA cm^−2^ higher than that of EG/(Co, Ni)Se_2_ and EG/CoSe-NC, and even tenfold that of EG/Ni_3_Se_2_ and commercial Ir/C’s current densities (6.32 and 6.15 mA cm^−2^) at the same potential. Notably, such a high OER performance of EG/(Co, Ni)Se_2_–NC hybrid overwhelmed that of commercial Ir/C and even most reported binary CoNi selenide-based OER electrocatalysts (Table S3).

The Tafel plot of EG/(Co, Ni)Se_2_–NC hybrid was further measured (Fig. 4c), where the EG/(Co, Ni)Se_2_–NC hybrid exhibited the most facile kinetics with the smallest Tafel slope of 73.3 mV dec^−1^, compared to the EG/(Co, Ni)Se_2_ (87.2 mV dec^−1^), EG/CoSe-NC (90.7 mV dec^−1^), EG/Ni_3_Se_2_ (230 mV dec^−1^), and commercial Ir/C (98.1 mV dec^−1^), suggesting a favorite reaction kinetic of EG/(Co, Ni)Se_2_–NC hybrid. Clearly, the EG/(Co, Ni)Se_2_–NC hybrid displayed a faster reaction rate than the EG/CoSe-NC, revealing that the incorporation of Ni species apparently boosted the OER electrocatalysis. Electrochemical impedance spectroscopy (EIS) offered an explanation to the facile kinetics and fast electron transfer process of EG/(Co, Ni)Se_2_–NC hybrid (Fig. 4d). The charge transfer resistance could be evaluated from the radius of each curve, in which a smaller radius indicated a less charge transfer resistance. The charge transfer resistance of EG/(Co, Ni)Se_2_–NC hybrid was the lowest among all the investigated samples, suggesting the facile electron transfer in the EG/(Co, Ni)Se_2_–NC. Especially, the semi-circle of EG/(Co, Ni)Se_2_–NC hybrid was evidently smaller than that of EG/CoSe_2_–NC, manifesting that the presence of Ni species in this hybrid could make the electron transfer process more quick and therefore contribute to a higher electro-catalyzing performance [1, 31]. Furthermore, the durability of EG/(Co, Ni)Se_2_–NC hybrid was examined via a chronoamperometric method at a constant current density of 20 mA cm^−2^ (inset of Fig. 4e). The potential of EG/(Co, Ni)Se_2_–NC hybrid only increased from 1.53 to 1.54 V after 10 h reaction, and the merely 0.65% increase in the potential indicated the excellent stability of EG/(Co, Ni)Se_2_–NC. Meanwhile, the polarization curves of EG/(Co, Ni)Se_2_–NC before and after 1000 CV cycles were compared, and little difference in both overpotential at 10 mA cm^−2^ (258 mV before OER and 249 mV after OER) and onset potential (1.491 V before OER and 1.505 V after OER) was observed, demonstrating its outstanding stability (Fig. 4e). Importantly, the EG/(Co, Ni)Se_2_–NC hybrid could be also coupled with solar energy for catalyzing water splitting (Fig. S4, S5), demonstrating the practical usefulness of EG/(Co, Ni)Se_2_–NC hybrid to convert sustainable solar energy to hydrogen power.

The electrochemically active surface area (ECSA) was further estimated by comparing the double-layer capacities (*C*_dl_) of selenized samples [22, 26]. A larger *C*_dl_ usually represents a larger ECSA with more exposed active sites. In Fig. 4f, the EG/(Co, Ni)Se_2_–NC hybrid exhibited the largest *C*_dl_ of 53.8 mF cm^−2^, which was greater than the 53.1 mF cm^−2^ for EG/(Co, Ni)Se_2_, 48.5 mF cm^−2^ for EG/CoSe-NC, and 45.5 mF cm^−2^ for EG/Ni_3_Se_2_, respectively. The highly exposed active sites could strongly accelerate the OER electrocatalysis and make the EG/(Co, Ni)Se_2_–NC the best electrocatalyst benefiting from the synergy between (Co, Ni)Se_2_ and N-doped carbon, as well as the modified electronic structure with quick electron transfer process provided by Ni species [5, 27, 32].

**Fig. 5:**
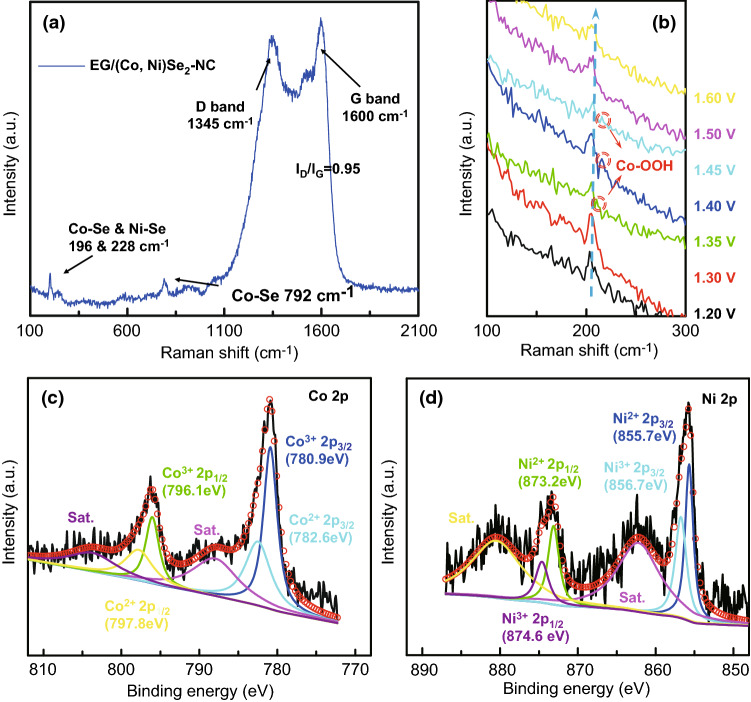
**a** Raman spectrum of EG/(Co, Ni)Se_2_–NC. **b** In situ electrochemical Raman spectra of EG/(Co, Ni)Se_2_–NC, potential ranges from 1.2 to 1.6 V. **c** High-resolution XPS spectrum of Co 2*p* in EG/(Co, Ni)Se_2_–NC after OER. **d** High-resolution XPS spectrum of Ni 2*p* in EG/(Co, Ni)Se_2_–NC after OER test

In situ electrochemical Raman spectroscopy was employed to further study the mechanism of EG/(Co, Ni)Se_2_–NC hybrid in catalyzing OER. In Fig. 5a, at the potential of 0 V, the Raman spectrum of EG/(Co, Ni)Se_2_–NC exhibited the peaks of Co–Se bonds and Ni-Se bonds located at 196 and 228 cm^−1^ in each instance [25, 28]. The peaks located at 1345 and 1600 cm^−1^ could be assigned to D and G bands of carbon with a ratio of *I*_D_/*I*_G_ being 0.95. At the potential of 1.2 V, the peak of Co–Se bonds was located at 202 cm^−1^. The increase in potential could lead to the shift of Raman peak from 202 to 206 cm^−1^ at the potential of 1.4 V; meanwhile, at this potential, another peak located at 210 cm^−1^ was also observed, which was well indexed to the Co–OOH species [5]. However, the intensity of this peak diminished severely after the potential of 1.5 V, on account for the generation of extremely active species, CoO_2_, under high voltages [27]. The ex situ XPS spectroscopy of EG/(Co, Ni)Se_2_–NC hybrid after electrochemical OER tests (Fig. 5c, d) revealed that the content of Co^3+^ species increased, while the content of Ni^3+^ species decreased. The rise of Co^3+^ species was a result of oxidation during catalyzing OER, indicating the Co species in the EG/(Co, Ni)Se_2_–NC participated in boosting OER as active sites. Notably, the diminishing of Ni^3+^ species and increasing of Ni^2+^ species in tested EG/(Co, Ni)Se_2_–NC hybrid presented the key role of introduced Ni species for tuning electronic structure [33], as the result of the electron binding energy of Ni decreasing integrally after OER test. Combining the in situ electrochemical Raman spectroscopy and ex situ XPS results, one can conclude that during the OER process, the original Co–Se bonds were oxidized to Co–OOH and CoO_2_ species, which are highly efficient active phases for electro-catalyzing OER, and the Ni species was participating in smoothing the electron transfer process [2, 8].

## Conclusions

In conclusion, a highly efficient hybrid OER electrocatalyst composed of core–shell-structured penroseit (Co, Ni)Se_2_ with N-doped carbon supported on EG was developed, of which the particle diameter of (Co, Ni)Se_2_ core was ~ 70 nm while the thickness of N-doped carbon shell was ~ 5 nm. Due to the synergistic effect between binary metals-based penroseit (Co, Ni)Se_2_ core and N-doped carbon shell, the EG/(Co, Ni)Se_2_–NC hybrid exhibited the superb electrocatalysis performance toward OER, with a low overpotential of 258 mV at the current density of 10 mA cm^−2^ and a small Tafel slope of 73.3 mV dec^−1^, which was superior to that of commercial Ir/C and most previously published binary CoNi selenide-based OER electrocatalysts. The combination of in situ electrochemical Raman spectroscopy and ex situ XPS measurement revealed that the high OER activity was attributed to the transformation from Co–Se to Co–OOH species during oxidation process with the help of facile electron transfer process from the reinforced electronic structure of introduced Ni species. It is believed that this unique core–shell-structured EG/(Co, Ni)Se_2_–NC hybrid could be an inspiration for proper design of binary transition metal selenides with N-doped carbon hybrid in energy-related applications such as lithium-ion battery, electrochemical urea oxidation, and supercapacitor.

## Electronic Supplementary Material

Below is the link to the electronic supplementary material.

Supplementary material 1 (PDF 835 kb)
