# Supplementary material for: Nitrogen-Doped Carbon-Encased Bimetallic Selenide for High-Performance Water Electrolysis
Source: Nanomicro Lett. 2019 Aug 8;11:67. doi: 10.1007/s40820-019-0299-4 (PMC7770840; doi:10.1007/s40820-019-0299-4)
Supplement: Supplementary file 1 — Supplementary material 1 (PDF 835 kb) [file 40820_2019_299_MOESM1_ESM.pdf]

Supporting Information for

Nitrogen-Doped Carbon Encased Bimetallic Selenide for High-Performance Water Electrolysis

Junhui Cao[1], Kexin Wang[1], Jiayi Chen[1], Chaojun Lei[1], Bin Yang[1], Zhongjian Li[1], Lecheng Lei[1], Yang Hou[1, 2, 3, *], Kostya (Ken) Ostrikov[4]

[1]Key Laboratory of Biomass Chemical Engineering of Ministry of Education, College of Chemical and Biological Engineering, Zhejiang University, Hangzhou 310058, People's Republic of China

[2]Ningbo Research Institute, Zhejiang University, Ningbo 315100, People's Republic of China

[3]Institute of Zhejiang University - Quzhou, 78 Jiuhua Boulevard North, Quzhou 324000, People's Republic of China

[4]School of Chemistry, Physics, and Mechanical Engineering, Queensland University of Technology Brisbane, QLD 4000, Australia

*Corresponding author. E-mail: yhou@zju.edu.cn (Yang Hou)

Supplementary Figures and Tables

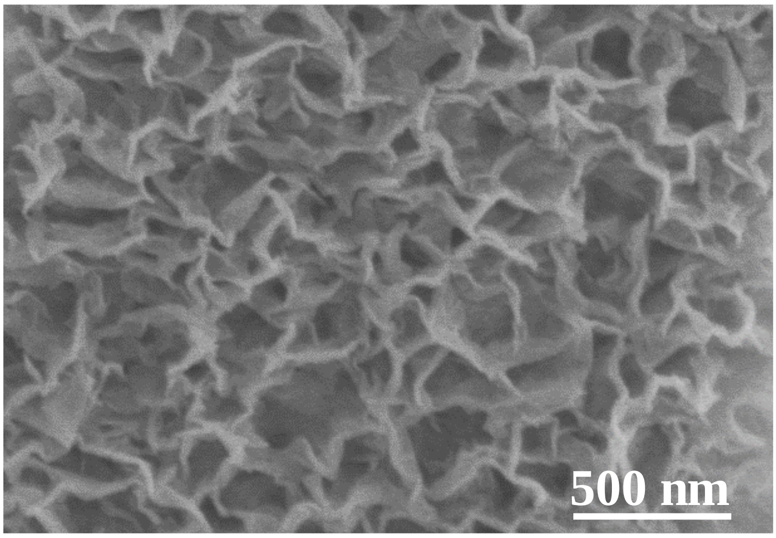

Fig. S1 FESEM image of EG/CoNi-LDH

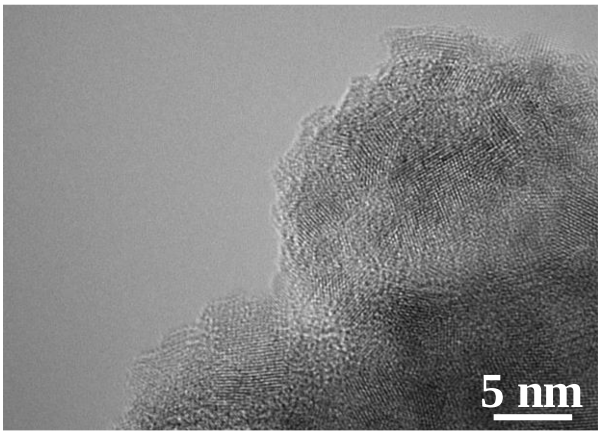

Fig. S2 TEM image of EG/CoNi-LDH

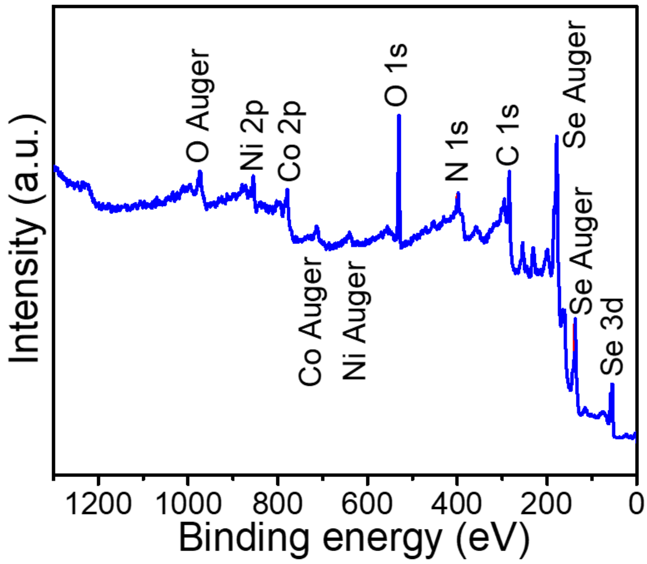

Fig. S3 XPS survey spectrum of EG/(Co, Ni)Se_2-NC

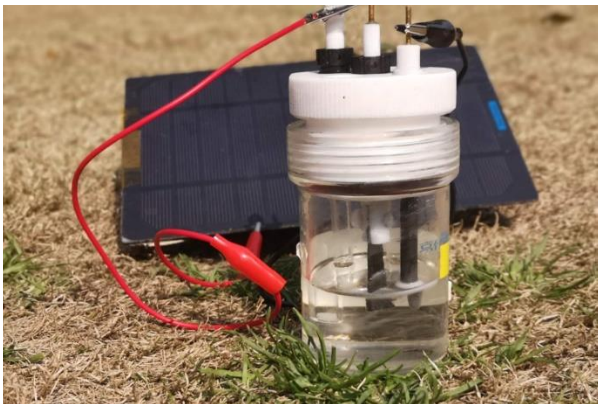

Fig. S4 Solar-energy driven water splitting with EG/(Co, Ni)Se_2-NC hybrid

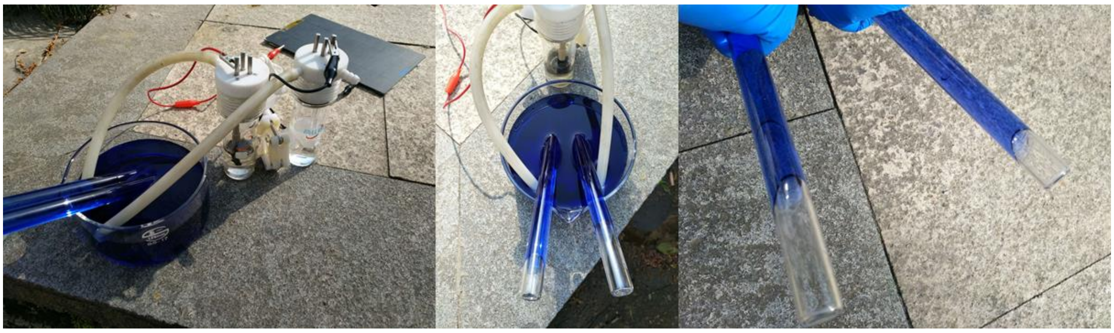

Fig. S5 Solar-energy driven water splitting with EG/(Co, Ni)Se_2-NC hybrid and product collecting system

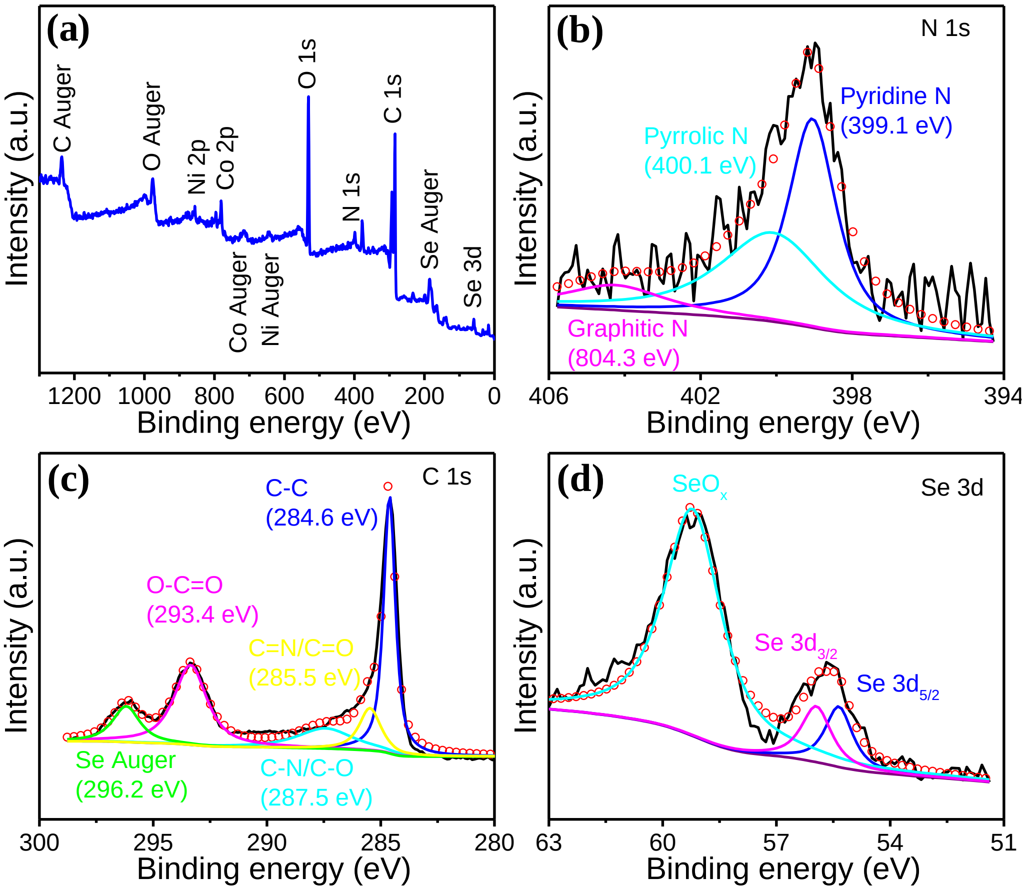

Fig. S6 XPS spectra of EG/(Co, Ni)Se_2-NC hybrid after OER test, **a** XPS survey spectrum; **b** high-resolution XPS spectrum of N 1s; **c** high-resolution XPS spectrum of C 1s; **d** high-resolution XPS spectrum of Se 3d

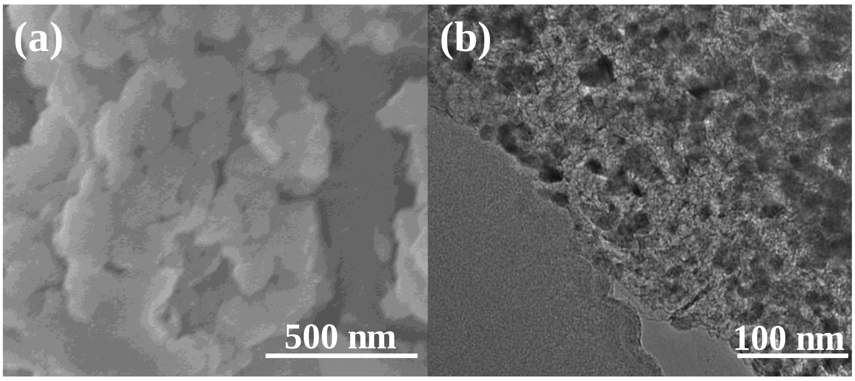

Fig. S7 a FESEM and **b** TEM images of EG/(Co, Ni)Se_2-NC hybrid after OER stability test

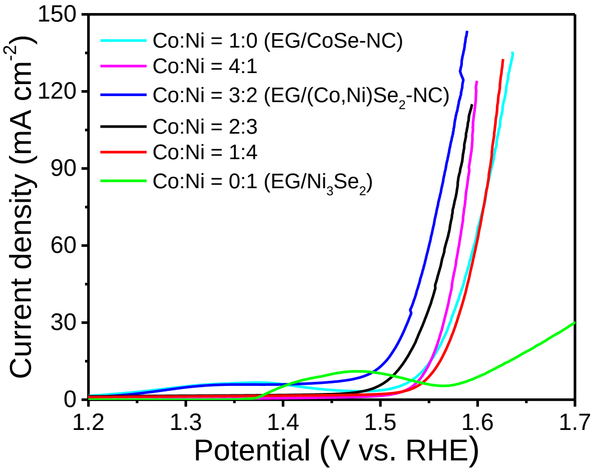

Fig. S8 Polarization curves of EG/(Co, Ni)Se_2-NC with different Co and Ni ratios, and EG/CoSe-NC and EG/Ni_3Se_2

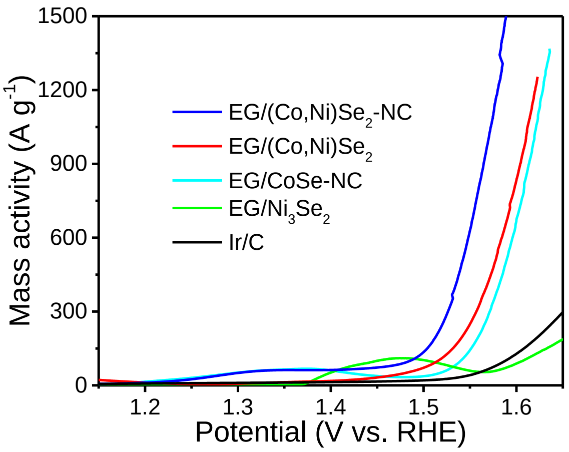

Fig. S9 Polarization curves of EG/(Co, Ni)Se_2-NC, EG/(Co, Ni)Se_2, EG/CoSe-NC, EG/Ni_3Se_2, and Ir/C with mass activity

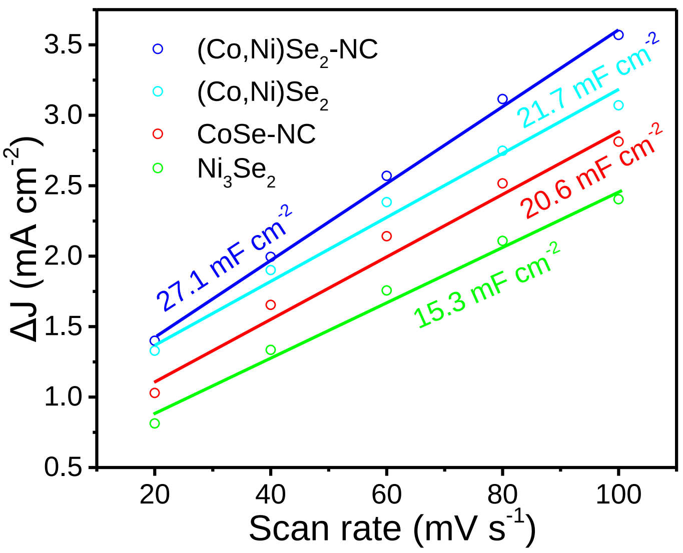

Fig. S10 C_{dl} plots of (Co, Ni)Se_2-NC, (Co, Ni)Se_2, CoSe-NC, and Ni_3Se_2

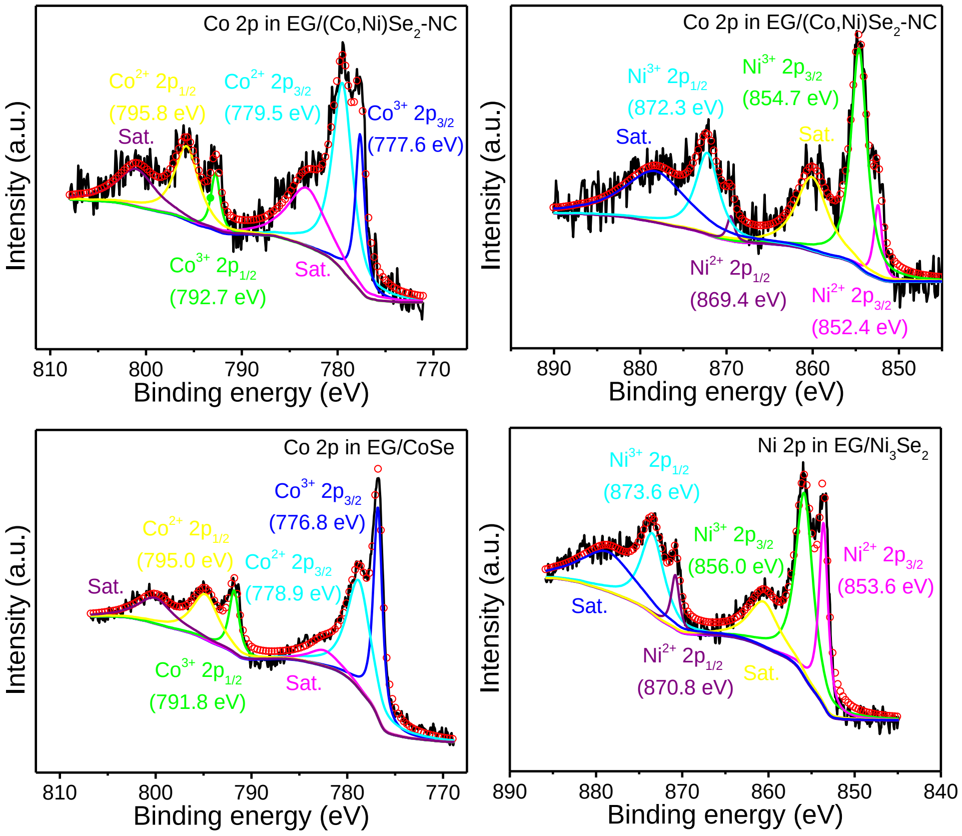

Fig. S11 High-resolution Co 2p XPS spectra from EG/CoSe and EG/(Co, Ni)Se_2-NC and high-resolution Ni 2p XPS spectra from EG/Ni_3Se_2 and EG/(Co, Ni)Se_2-NC

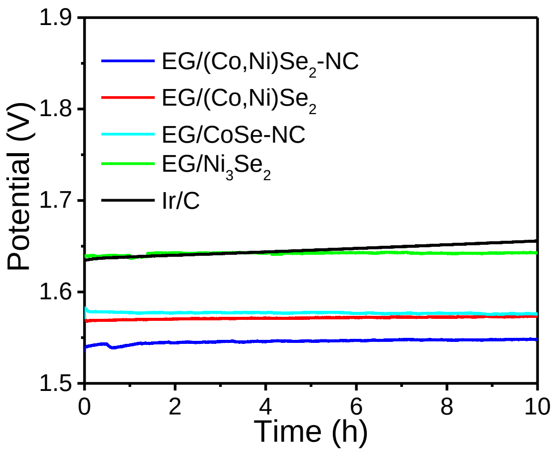

Fig. S12 Chronoamperometric *E*-*t* plot at 20 mA cm^{-2} of EG/(Co, Ni)Se_2-NC hybrid, EG/(Co, Ni)Se_2, EG/CoSe-NC, EG/Ni_3Se_2 and commercial Ir/C catalyst

Table S1 Elemental composition of EG/(Co, Ni)Se_2-NC via EDX measurements

Element	Line	Intensity (c/s)	Atomic %	Conc.	Units	Error 2-sig	
C	Ka	1.57	4.41	0.850	wt%	0.204	
N	Ka	0.27	0.635	0.143	wt%	1.037	
O	Ka	10.83	9.015	2.318	wt%	0.580	
Co	Ka	30.40	18.798	17.822	wt%	1.362	
Ni	Ka	36.40	19.815	18.786	wt%	1.620	
Se	Ka	9.95	47.327	60.081	wt%	12.339	
			100.000	100.000	wt%		Total

Table S2 Elemental composition of EG/(Co, Ni)Se_2-NC via XPS measurements

	Height (CPS)	FWHM (eV)	Area (P) CPS (eV)	Atomic (%)
N 1s	566.4	-0.02	8834.91	0.44
C 1s	43555.82	1.29	72400.52	64.27
Co 2p	4655.47	1.11	42550.71	6.02
Ni 2p	3044.29	3.5	14041.36	6.94
Se 3d	7214.71	1.8	25341.37	16.51
O 1s	16730.05	2.71	52401.86	15.82

Table S3 Comparison of OER performance of EG/(Co, Ni)Se_2-NC with other representative CoNi selenides based OER electrocatalysts

Author	Catalyst	Current density J	Potential at the corresponding J (vs. RHE)	Electrolyte
**This work**	**EG/(Co, Ni)Se_2-NC**	**10 mA cm^{-2}**	**1.49 V**	**1.0 M KOH**
ACS Appl. Mater. Interfaces, 2019, 11, 8106	(Co,Ni)Se_2@NiFe LDH	10 mA cm^{-2}	1.51 V	1.0 M KOH
Mater. Lett., 2019, 235, 53	$Fe_{0.08}Co_{0.09}Ni_{0.83}Se_2$	10 mA cm^{-2}	1.50 V	1.0 M KOH
Nanoscale, 2018, 10, 22012	Co-Sn-Se,	10 mA cm^{-2}	1.53V	1.0 M KOH
J. Mater. Chem. A, 2018, 6, 18641	$NiCoSe_2$@NiO@CoNi_2S_4@CoS_2/NF	30 mA cm^{-2}	1.54 V	1.0 M KOH
Electrochimica Acta, 2018, 265, 577	FCS@N-CT	10 mA cm^{-2}	1.56 V	1.0 M KOH
J. Mater. Chem. A, 2018, 6, 5999	Co LDH UTNSs	10 mA cm^{-2}	1.56 V	1.0 M KOH
ACS Sustain. Chem. Eng., 2018, 6, 10952	Y-S Ni-Co-Se/CFP	10 mA cm^{-2}	1.53 V	1.0 M KOH
J. Mater. Chem. A, 2018, 6, 7585	$(Ni,Co)_{0.85}Se$ NSAs	20 mA cm^{-2}	1.52 V	1.0 M KOH
Chem. Commun., 2017, 53, 5412	$CoNi_2Se_4$	10 mA cm^{-2}	1.39V	1.0 M KOH
ACS Catal., 2017, 7, 6394	(Ni,Co)Se_2-GA	10 mA cm^{-2}	1.48 V	1.0 M KOH
Electrochimica Acta, 2017, 250, 167	(Ni,Co)Se_2/C-HRD	10 mA cm^{-2}	1.48 V	1.0 M KOH
Adv. Funct. Mater., 2016, 26, 4661	$NiCo_2S_4$ NW/NF	10 mA cm^{-2}	1.49 V	1.0 M KOH
Energy Environ. Sci., 2016, 9, 1771	Ni_3Se_2	10 mA cm^{-2}	1.54 V	0.3 M KOH
